# Case report: Diagnostic trap: metastatic endometrial stromal sarcoma with breast metastasis

**DOI:** 10.3389/fonc.2024.1465052

**Published:** 2024-10-15

**Authors:** Xiaoxue Tian, Shuai Luo, Ting Xu, Jinjing Wang

**Affiliations:** Department of Pathology, Affiliated Hospital of Zunyi Medical University, Zunyi, Guizhou, China

**Keywords:** endometrial stromal sarcoma, metastasis, mammary gland, diagnosis, treatment

## Abstract

**Background:**

Endometrial stromal sarcoma (ESS) is a rare type of uterine malignancy typically classified into low-grade ESS (LG-ESS) and high-grade ESS. LG-ESS is characterized by low malignancy and limited metastasis, primarily to the lungs. Metastasis of the breast is extremely rare, posing significant challenges in clinical diagnosis and treatment.

**Case demonstration:**

A 33-year-old female with a history of two cesarean sections was diagnosed with uterine LG-ESS five months prior. She was admitted for the excision of a left breast mass discovered during a routine examination. A histopathological biopsy confirmed the mass as a breast metastasis of LG-ESS. Postoperatively, she underwent radiotherapy and chemotherapy at a cancer hospital. She has been followed up on for two years with no recurrence.

**Conclusions:**

ESS with breast metastasis is extremely rare. The morphological features of ESS with breast metastasis can resemble mesenchymal and sex cord-stromal tumors, complicating imaging and pathological diagnosis, especially if there is no known history of uterine ESS. This study highlights the clinicopathological features of LG-ESS with breast metastasis, including clinical manifestations, imaging features, histopathology, immunohistochemistry, molecular genetic features, and treatment prognosis. It aims to provide new insights for the clinical diagnosis and treatment of ESS with breast metastasis.

## 1 Background

Endometrial-stromal tumors are rare uterine tumors. In 2020, the World Health Organization classified these tumors into endometrial stromal nodules (ESN), low-grade endometrial stromal sarcoma (LG-ESS), high-grade endometrial stromal sarcoma (HG-ESS), and undifferentiated uterine sarcoma (UUS) (1). Among these, LG-ESS accounts for less than 1% of all uterine malignancies and only 0.2% of female reproductive tract malignancies, making it second only to leiomyoma in frequency. LG-ESS is associated with partially detectable characteristic molecular genetic changes.

Patients with LG-ESS generally have a better prognosis compared to those with HG-ESS (2). However, metastasis can still occur. The lungs are the common site for LG-ESS metastasis, but metastases to the pelvic cavity, abdomen, bone, and atrium have also been reported. Breast metastasis is extremely rare, with only three reported cases.

Due to the rarity of endometrial stromal sarcoma (ESS) in breast metastasis, it is often overlooked, especially in the absence of a prior history of ESS. This poses significant diagnostic challenges. This study reports a case of LG-ESS with breast metastasis and reviews relevant literature. It emphasizes the importance of considering a uterine origin when diagnosing female spindle cell tumors and aims to enhance the understanding of the diagnosis and treatment of this disease.

## 2 Case demonstration

A 33-year-old female patient with a history of two cesarean sections was admitted to the hospital due to a pelvic mass detected more than a year ago and irregular vaginal bleeding for over two months. She experienced intermittent abdominal pain, dizziness, and tinnitus. During the physical examination, an 11-cm longitudinal surgical scar was observed. Her abdomen was distended, resembling the size of a seven-month pregnancy, and she exhibited tenderness without rebound pain or muscle tension. Gynecological examination revealed a large, tough, immobile lump, reaching three transverse fingers below the xiphoid process, with mild tenderness. The uterus and bilateral adnexa were not clearly discernible, and there were no obvious abnormalities in the vulva and vagina.

After admission, a vaginal color ultrasound examination was performed. The uterine body contour was unclear, and the cervix was small with a uniform echo. Several solid hypoechoic masses of varying sizes were observed from three transverse fingers below the cervix to the xiphoid process, some of which had fused together, measuring approximately 15 x 10.1 x 14.3 cm. The internal echo was uneven, with a few fluid areas and striped blood flow signals (RI 0.44). The findings suggested a solid abdominal space-occupying lesion, possibly a uterine sarcoma. A retroperitoneal tumor was also considered, and further examination was recommended.

**Figure 1:**
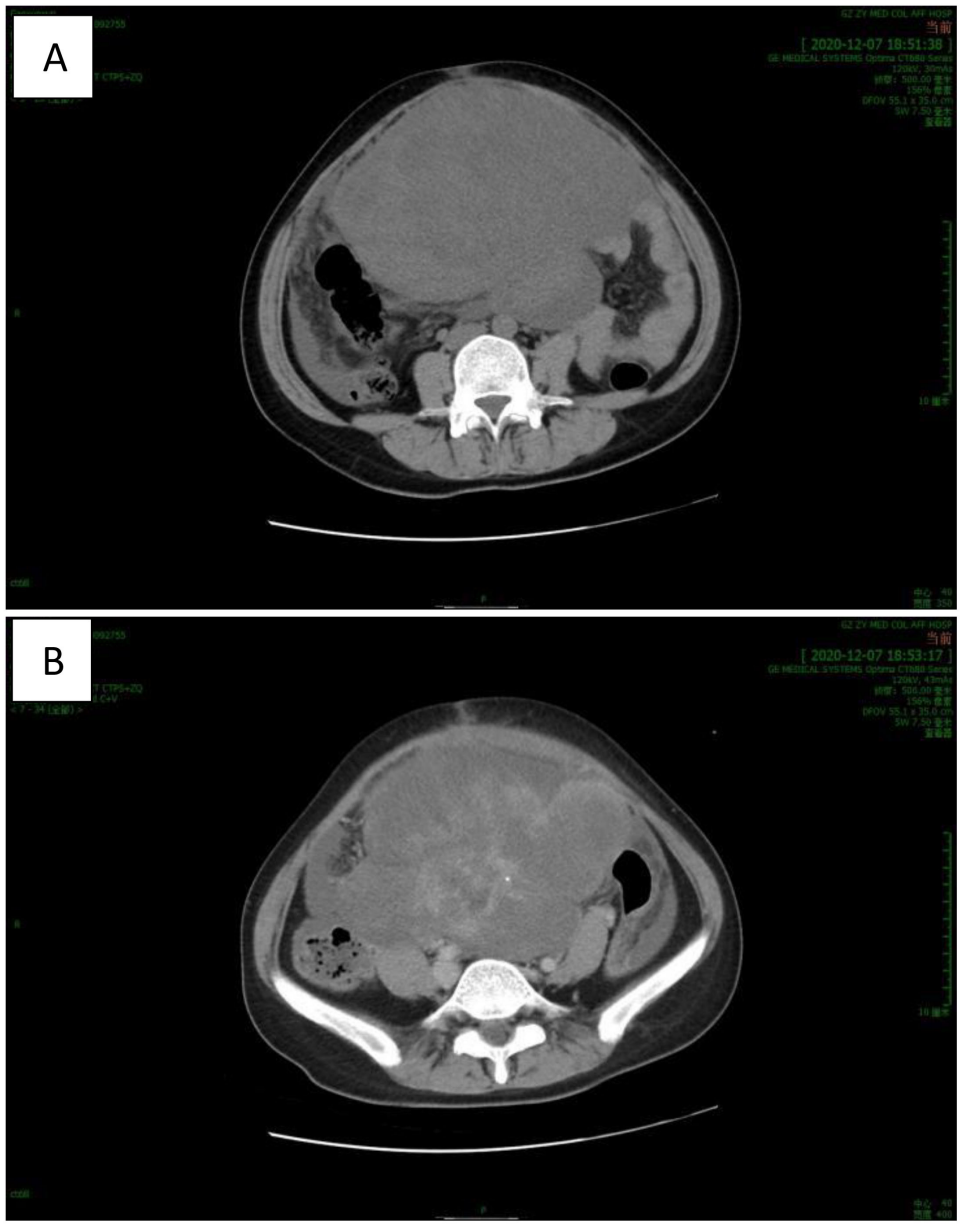
CT revealed a huge mass in the middle and lower abdomen with uneven density and septation. [**(A)** plain scan, **(B)** enhanced scan].

Abdominal computed tomography (CT) (**Figure 1**) revealed a huge mass in the middle and lower abdomen, measuring approximately 178 x 143 x 180 mm, with uneven density and septation. The mass had unclear boundaries with the right adnexal area, adjacent uterus, and peritoneum and exhibited uneven, moderate enhancement. The uterus was significantly enlarged with multiple nodules and masses, showing uneven density, spotty and nodular calcification, uneven enhancement, and fluid density shadows in the uterine cavity. The liver, spleen, gallbladder, pancreas, kidneys, and bladder appeared normal in size, morphology, and density. No enlarged lymph nodes were seen in the abdominal, pelvic, or retroperitoneal regions, and there was minimal fluid accumulation in the abdominal and pelvic cavities. The findings indicated a large space-occupying lesion in the middle and lower abdominal cavities, multiple uterine nodules and masses, and a small amount of fluid in the abdominal and pelvic cavities. IOTA: M(presence of solid mass with irregularity, with ascites, solid mass with a maximum diameter of more than 100 mm, and abundant blood flow signal).

Auxiliary examination results included: human chorionic gonadotropin (HCG): negative; Gly antigens 125: 343.7 U/mL. No other obvious abnormalities were detected.

After admission, clinicians considered the possibility of a uterine leiomyoma and performed an open myomectomy with pelvic adhesion lysis. Intraoperatively, they observed peritoneal congestion and thickening, with approximately 500 mL of dark red bloody ascites. The uterus appeared congested with multiple prominent tumors on the surface, the largest measuring approximately 15 x 14 x 12 cm3, lacking a complete capsule, and appearing brittle. The uterus, right fallopian tube, and ovary were densely adhered and hard, with multiple rice-grain-sized nodules on the surface. No obvious abnormalities were observed in the left adnexa, and bilateral uterosacral ligaments were free of palpable nodules or other abnormalities.

The histopathological biopsy was sent postoperatively. Macroscopically, the entire uterus measured 17 x 16.5 x 8 cm. The uterine mucosal surface, muscle wall, and serosa surface exhibited numerous gray nodules. the boundary of the nodule was poorly defined. The cut surface was gray, mass-like, resembling a worm or fish, with a diameter ranging from 3.5 to 12.5 cm, some protruding through the serous membrane with clear boundaries.

**Figure 2:**
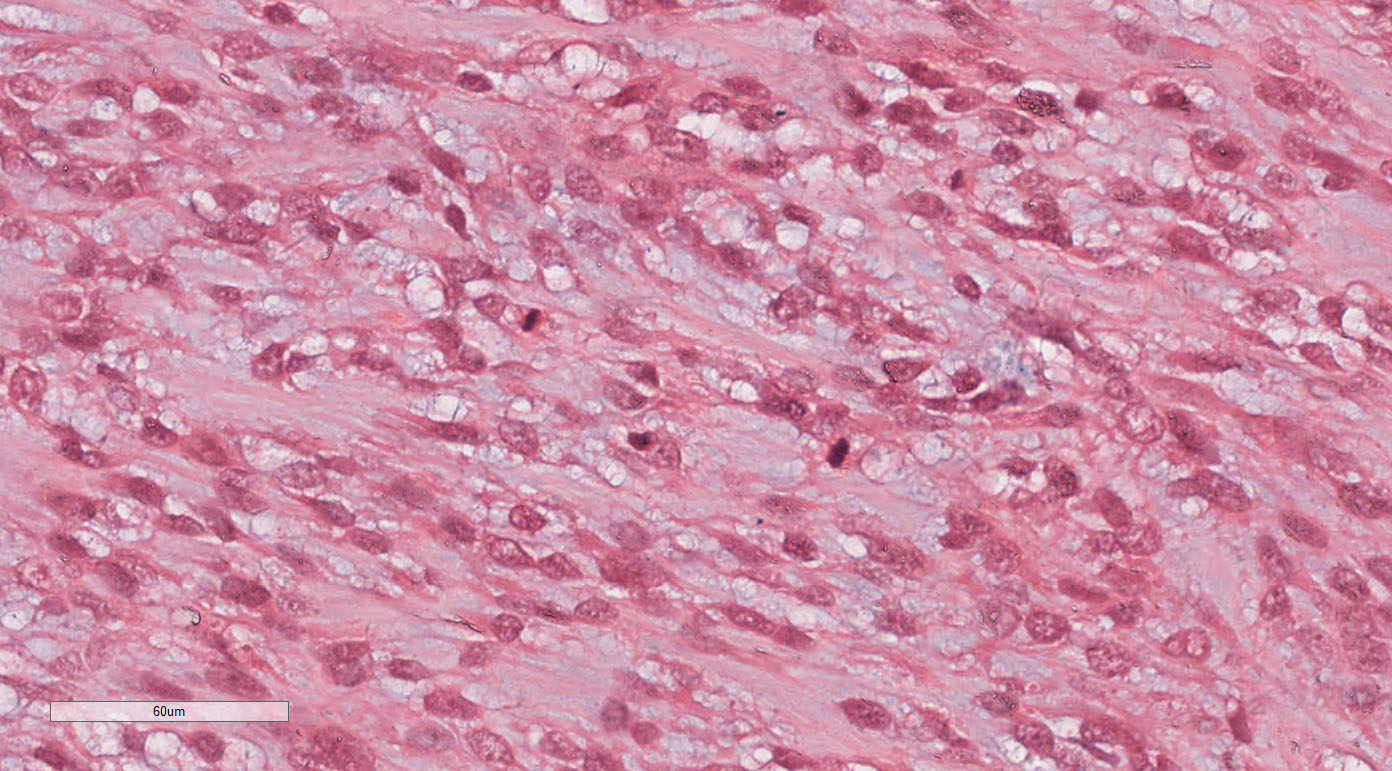
LG-ESS of the uterus: the nuclei appeared uniform, ranging from oval to spindle-shaped, with minimal atypia and scant cytoplasm. Nuclear division was rare. H&E ×400.

Under low magnification, irregular and dense tumor cells infiltrated the myometrium in an island- or tongue-like pattern, with visible vascular infiltration. At high magnification (**Figure 2**), the nuclei appeared uniform, ranging from oval to spindle-shaped, with minimal atypia and scant cytoplasm. Nuclear division was rare.

**Figure 3:**
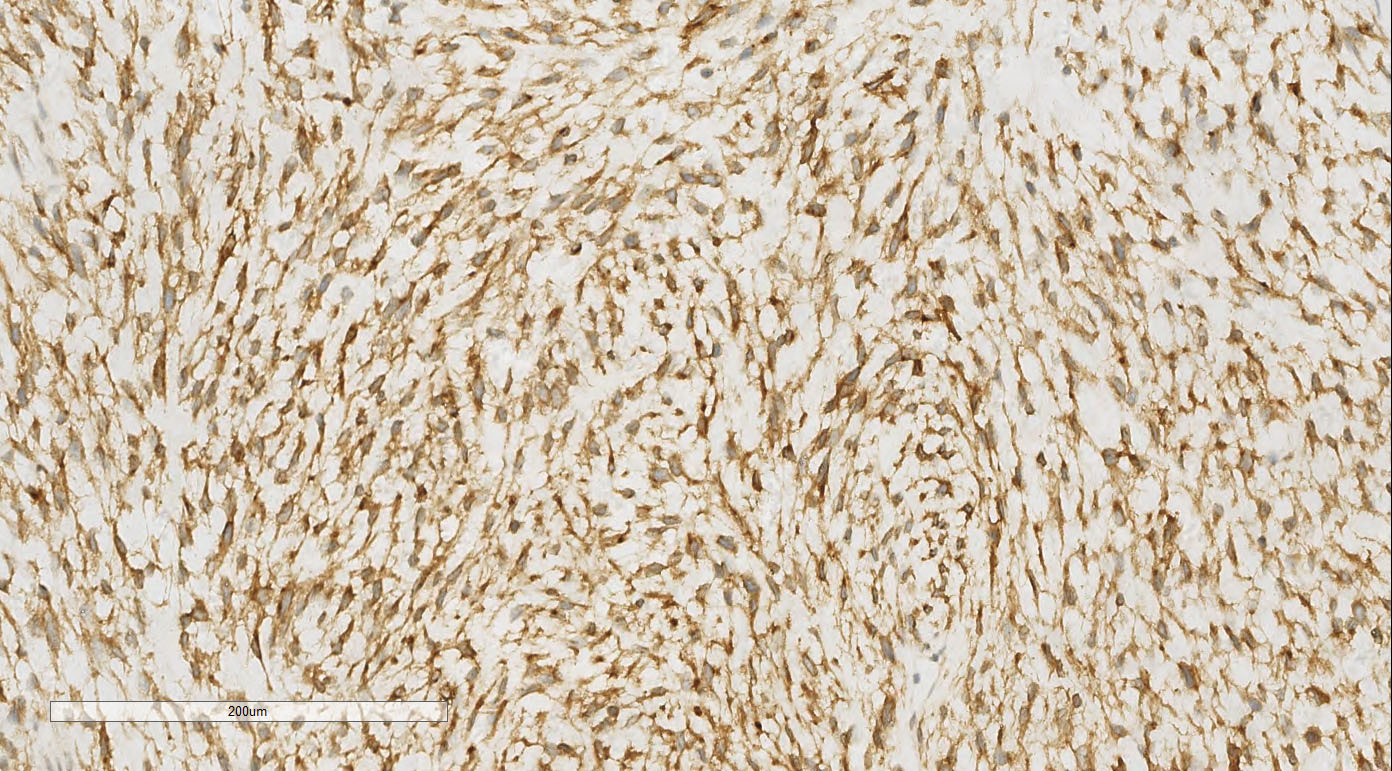
LG-ESS of the uterus: Immunohistochemistry reveals a tumor cells CD10 (+). EnVision, ×200.

The immunohistochemical (IHC) results indicated the presence of spindle tumor cells with the following markers: vimentin (+), CD10 (+), cyclin D1 (+) (**Figure 3**), Ki-67 (40% +), calponin (-), CD34 (-), CK (-), Desmin (-), estrogen receptor (ER; -), progesterone receptor (PR; -), S100 (-), and smooth muscle actin (SMA; -). The final pathological diagnosis was LG-ESS of the uterus. After confirming the diagnosis, she was discharged for adjuvant treatment.

Five months later, the patient was readmitted for the evaluation of a newly discovered left breast mass. She had inadvertently found a left breast mass without tenderness, nipple discharge, or hemorrhage. She reported no chills, fever, fatigue, night sweats, chest distress, chest pain, or breathing difficulties. A physical examination revealed bilateral breast symmetry with no nipple retraction. The local skin showed no swelling or rupture, and there was a surgical scar. A palpable lump in the left breast measured about 3 cm in diameter, was tough with good mobility, had clear boundaries, a smooth surface, and no tenderness or adhesion to the chest wall or skin. A slightly hard nodule in the right breast, about 0.5 cm in diameter, also had good mobility. No enlarged lymph nodes were palpable in the axilla, supraclavicular, or subclavicular areas. Auxiliary examination showed a normal sugar antigen 25 level of 7.4 U/mL.

The bilateral breast-color ultrasound showed a lesion in the left breast located approximately 30 mm from the nipple at the 4 o’clock position. This lesion had a clear boundary, was primarily fluid-filled, had poor sound transmission, an enhanced posterior echo, and no obvious blood flow signal. Another hypoechoic nodule was observed about 67 mm from the nipple at the 9 o’clock position, with uneven internal echo, strong light spots, and an acoustic shadow, but no obvious blood flow signal within the nodule. The findings suggested local thickening of bilateral mammary gland tissue and hypoechoic nodules in both breasts, with milk stasis needing to be excluded.

Further ultrasound imaging revealed local thickening and disordered echoes in the bilateral mammary gland tissue, with slight breast duct expansion. In the left breast, approximately 25 mm from the nipple, a mixed echo nodule measuring about 28 x 19 mm was observed. This nodule had a clear boundary, regular morphology, was primarily fluid-filled with poor sound transmission, and showed enhanced posterior echo with visible linear blood flow signals. In the right breast, approximately 30 mm from the nipple, a nodule measuring about 44 mm was found. This nodule had an unclear boundary, irregular morphology, an uneven internal echo, an acoustic shadow, and no obvious blood flow signals. Ultrasound imaging after injection of a contrast agent showed low enhancement in the arterial and parenchymal phases of the hypoechoic region in the right breast, with perfusion and regression starting almost simultaneously with the normal tissue. After contrast injection, the boundary remained unclear, the shape was irregular, and the perfusion range was about 43 mm. In the left breast, the cystic nodular artery showed slightly higher enhancement during the parenchymal phase, with an unclear boundary, a regular shape, and no abnormal contrast perfusion in the surrounding tissue. Considerations included the local thickening of bilateral mammary gland tissue. The left cystic nodule was classified as BI-RADS 4b, suggesting fibroadenoma with cystic changes combined with ultrasound contrast. The hypoechoic region in the right breast was also classified as BI-RADS 4b, indicating possible adenopathy or breast carcinoma, which needs to be ruled out.

The double breast masses were initially suspected to be fibromas or breast cancer. Subsequently, the patient underwent “left breast mass resection + fascia valvuloplasty + minimally invasive rotation resection of the right breast mass under color ultrasound guidance. “During the operation, a mass measuring approximately 4 x 3 cm. was identified in the left breast gland between the 4 o’clock and 6 o’clock positions. It had a complete capsule, tough consistency, clear boundaries, and good mobility. Simultaneously, the right breast mass located at the 9 o’clock position was targeted under color ultrasound guidance. A 0.5-cm incision was made, and the anchor needle was placed under the mass. The rotational needle was then activated under ultrasound guidance to completely excise the right breast lesion.

Histopathological biopsies were performed postoperatively. A gross examination revealed the following findings: In the left breast mass, the tissue appeared gray-red, measuring 3 x 2.5 x 2 cm, with a grayish section displaying a solid mass enclosed within an intact capsule and evidence of local bleeding. In the right breast mass, the tissue appeared broken, with gray-red and gray-yellow areas, measuring 1.5 x 1 x 0.5 cm, and a gray-white section.

**Figure 4:**
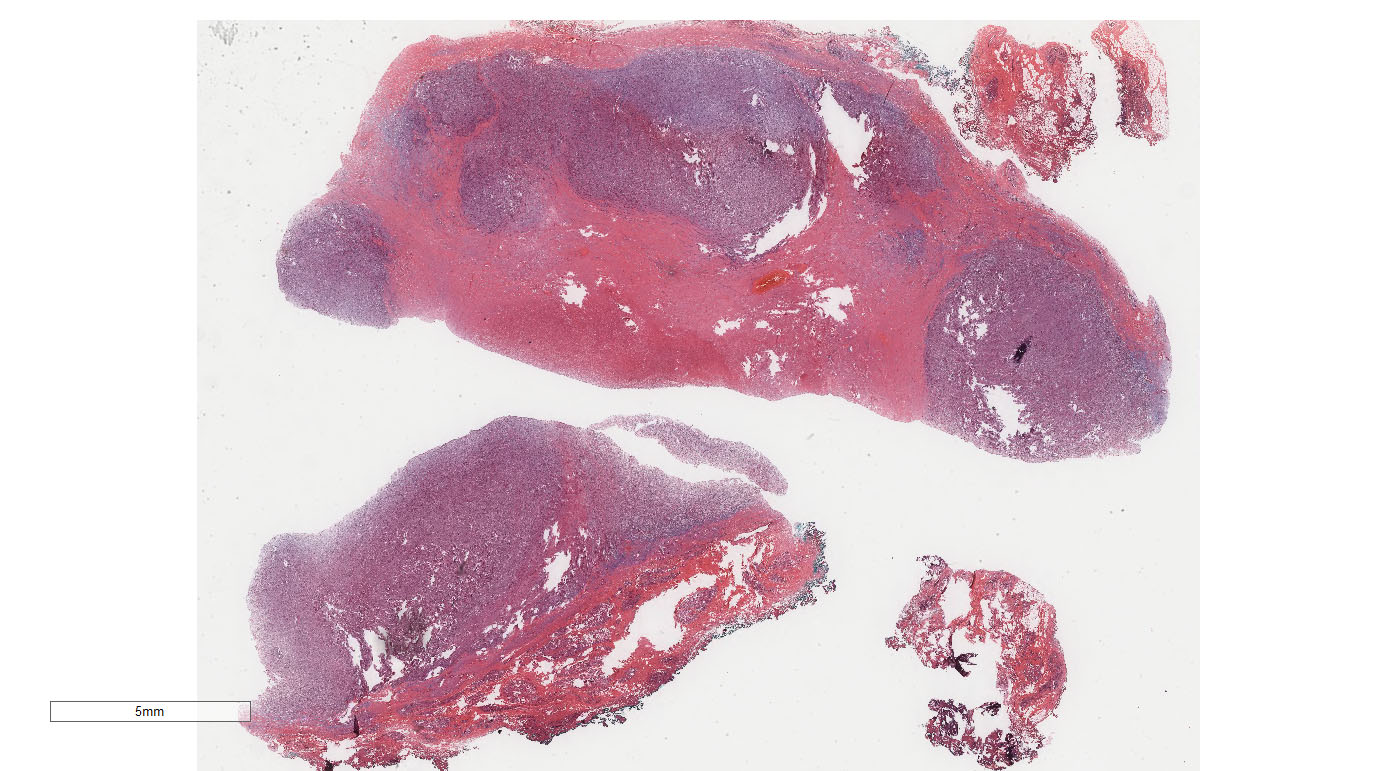
Breast metastases: Low power microscopy showed that nodular tumors were observed within the breast parenchyma, showing clear borders.H&E ×4.

**Figure 5:**
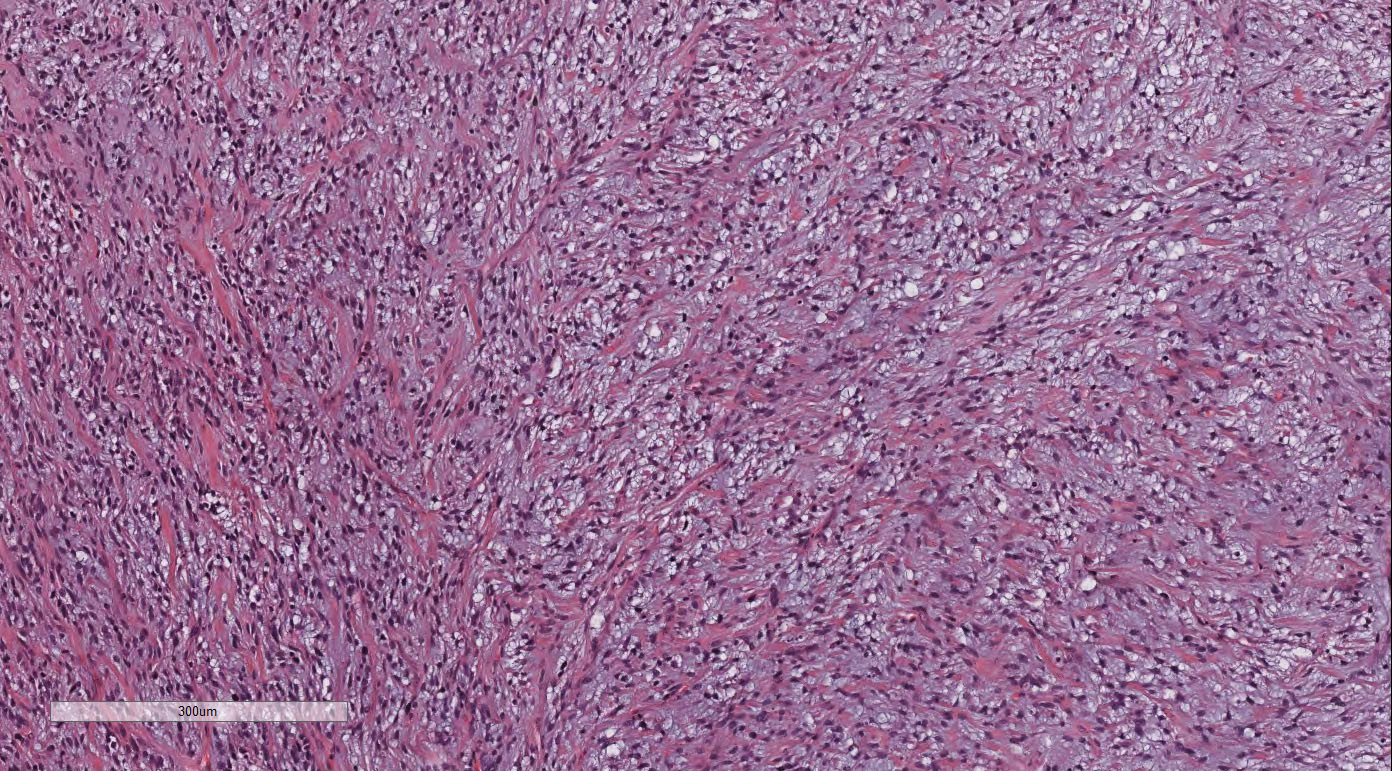
Breast metastases: The tumor cells were small, predominantly oval or spindle-shaped, with scant cytoplasm. H&E ×200.

**Figure 6:**
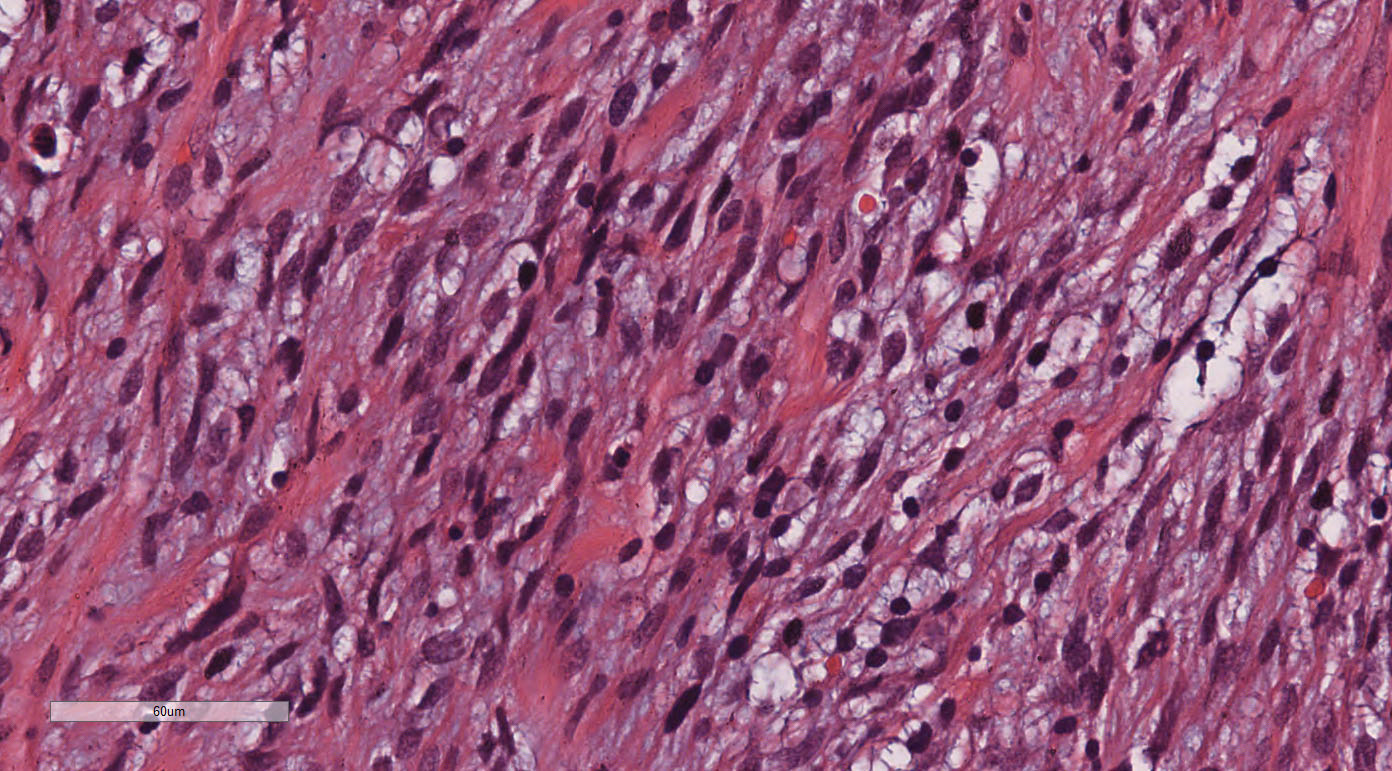
Breast metastases: Nuclear chromatin was uniform with minimal atypia, and there were rare instances of nuclear division. H&E ×400.

Under low-magnification microscopy (**Figure 4**), nodular tumors were observed within the breast parenchyma, showing clear borders. The tumor cells were small, predominantly oval or spindle-shaped, with scant cytoplasm(**Figure 5**), densely packed, and uniform in appearance. Nuclear chromatin was uniform with minimal atypia, and there were rare instances of nuclear division(**Figure 6**). The histomorphology closely resembled that of previous cases of ESS.

**Figure 7:**
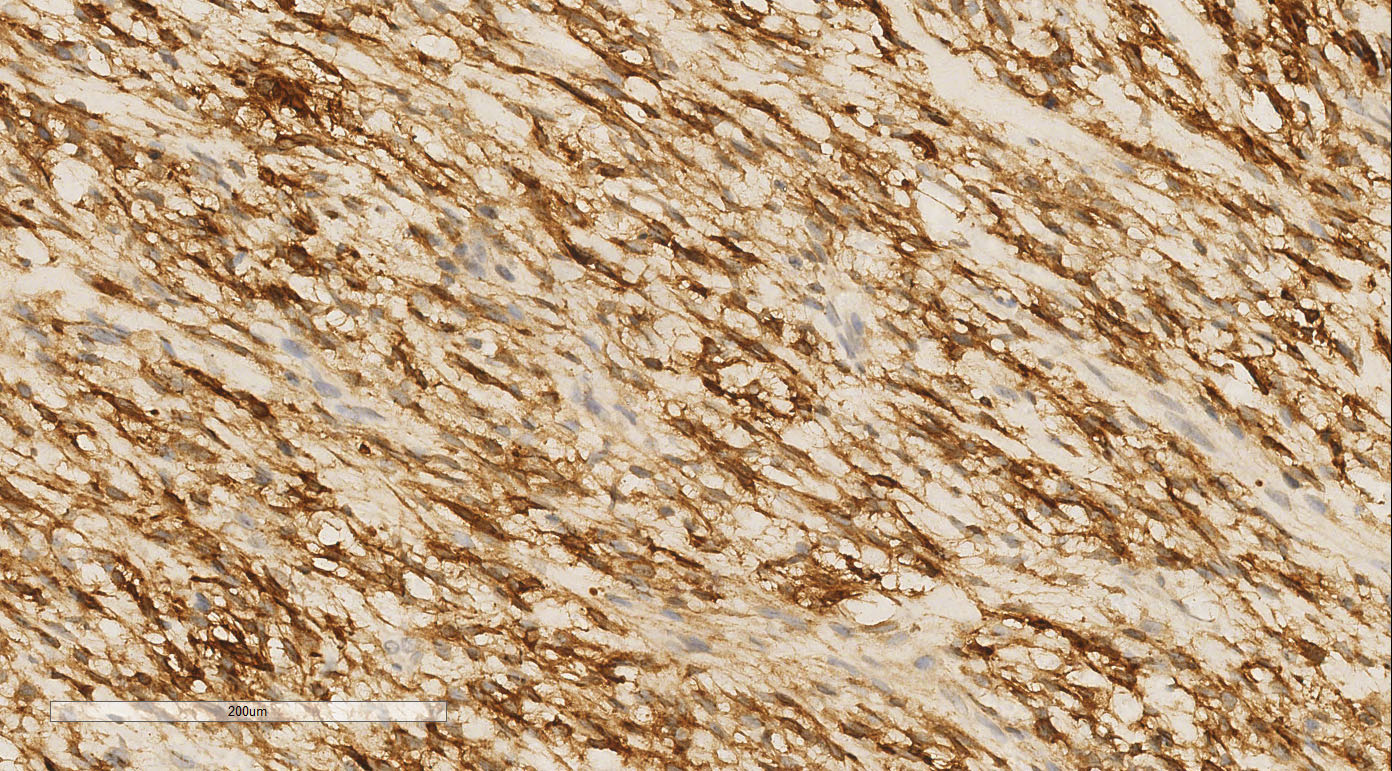
Breast metastases: Immunohistochemistry reveals a tumor cells CD10 (+). EnVision, ×200.

**Figure 8:**
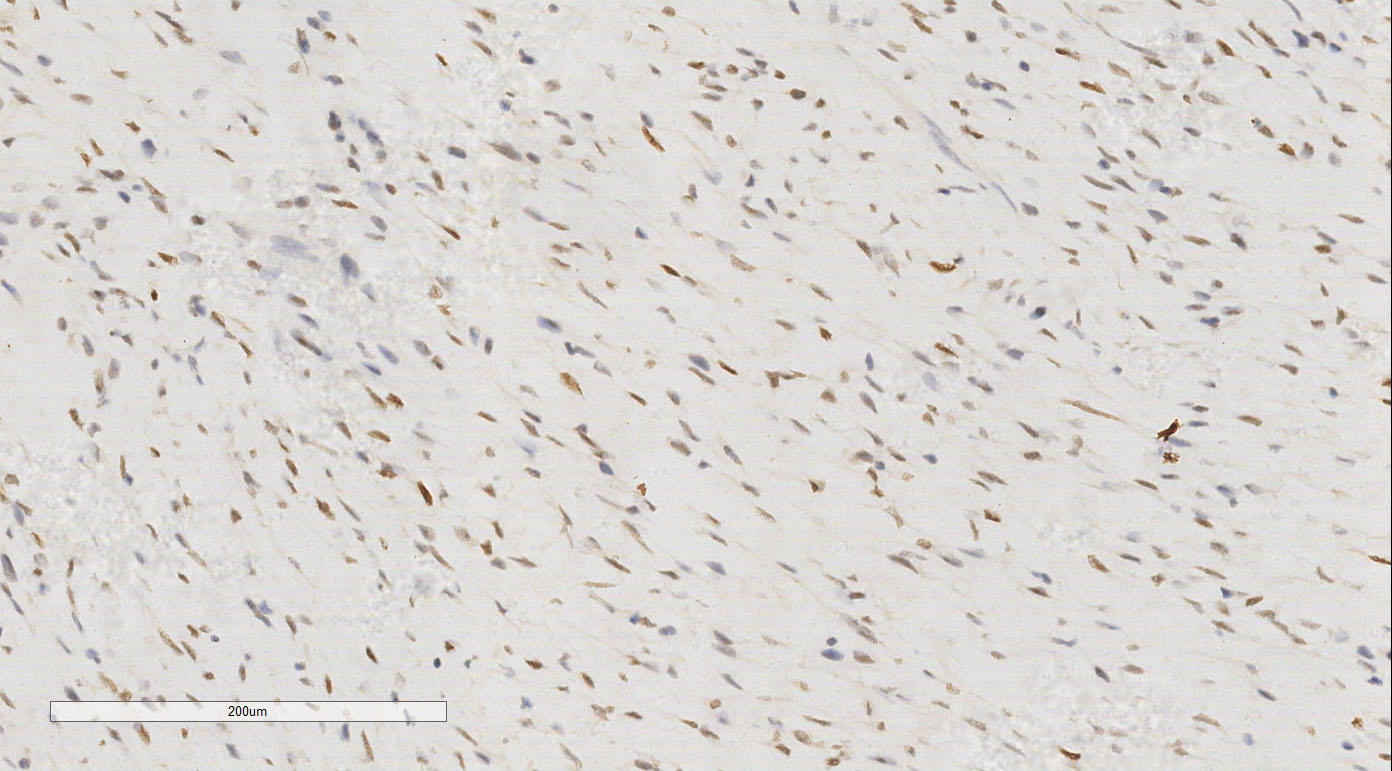
Breast metastases: Immunohistochemistry reveals a tumor cells cyclin D1 (+). EnVision, ×200.

IHC analysis revealed the following tumor cell markers: vimentin (+), CD10 (+) (**Figure 7**), cyclinD1 (+) (**Figure 8**), CK (+, locally scattered minority), S100 (+, scattered weak), SMA (+/-, scattered), PR (+/-, partially scattered), CD117 (+/-, localized in minority), Ki-67 (+, 40%), ER (-), H-Caldesmon (-), CD163 (-), CD34 (-), CK5/6 (-), Desmin (-), HMB45 (-), p63 (-), and STAT6 (-).

Based on the characteristic histological morphology and IHC results, combined with the patient’s previous history of ESS, the final diagnosis was ESS left breast metastasis.

The patient was transferred to a cancer hospital for chemoradiotherapy and has been followed up for two years without recurrence.

## 3 Discussion

ESS is a rare type of uterine malignant tumor, accounting for only 0.2% of primary uterine malignancies (3) and comprising 15% of uterine sarcomas (4). ESS is typically classified into LG-ESS and HG-ESS categories. LG-ESS involves the proliferation of endometrial stromal cells with potential infiltration of the myometrium and lymphatic vessels. It is characterized by low malignant potential, rare metastases, and predominantly lung metastases. Breast metastasis is extremely rare, posing significant challenges in clinical diagnosis and management.

This paper aims to explore the clinicopathological features of LG-ESS with breast metastasis, emphasizing clinical manifestations, imaging features, histopathology, IHC, molecular genetic features, and treatment outcomes in cases of ESS with breast metastasis.

A search of the PubMed database using the terms “ESS” and “breast” identified relevant literature. The inclusion criteria encompassed case reports or case series of patients with primary uterine ESS and ESS with breast metastasis. The exclusion criteria included cases of extrauterine primary ESS. The search yielded three relevant case reports documenting ESS with metastasis (**Table 1**).

**Table 1:** A literature review of ESS with breast metastasis.

Time	Author	Number of cases	Age	Diameter(cm)	Position	Grade	Gene mutation	The time of the transfer	Therapeutic regimen
2002	Günhan-Bilgen (5)	1	56	2.5	In the lower outer quadrant of the right breast	low-grade	none	17 years	None
2023	Amolika Namjoshi (6)	1	47	2.4	In the left breast, 9 o’clock position	high-grade	*BCOR* disruption and loss of one allele in 74.5% of nuclei.	None	None
2023	Ashlyn Fong (7)	1	59	1	In the right breast, 12 o’clock position	high-grade	BCOR rearrangement	Few months	Docetaxel and gemcitabine chemotherapy
	Our case	1	33	3	In the left breast, 4 o’clock position	low-grade	none	Five months	Surgery and chemotherapy

ESS, endometrial stromal sarcoma.

### 3.1 Clinical feature of ESS with breast metastasis

#### 3.1.1 Clinical characteristics

ESS with breast metastasis affects exclusively females, with a mean age of 48.75 years and a mean tumor diameter of 2.22 cm. Two patients involved the left breast, and two patients involved the right breast. All four patients had a history of ESS, and the breast masses were discovered incidentally or during a physical examination. These masses were typically painless, with borders that were round, irregular, or lobulated. The case reported here represents the youngest patient with the largest diameter among patients with ESS with breast metastasis.

#### 3.1.2 Time of metastasis

In two out of four patients with ESS with metastasis, breast metastasis occurred within months after the initial diagnosis of ESS. In another patient, the metastasis occurred as late as 17 years after the initial diagnosis.

#### 3.1.3 Imaging features

X-ray and ultrasound examinations revealed distinctive features in ESS with metastasis. In two patients, the masses showed irregular patterns resembling the myometrial invasion seen in LG-ESS, suggesting infiltration into the breast stroma, possibly via permeation from the primary tumor site. In the current patient, imaging showed mixed echogenic nodules with clear borders, predominantly fluid-filled, poor sound transmission, enhanced posterior echo, and no detectable blood flow signals.

Based on a literature review (8), metastatic breast cancer typically presents as single or multiple well-defined round or oval hypoechoic nodules without irregular borders, calcifications, or secondary skin or nipple changes suggestive of malignancy. Exceptions include cases of ovarian cancer, hepatocellular carcinoma, and gastrointestinal cancer breast metastasis, which may exhibit microcalcifications. Therefore, the absence of microcalcifications can be crucial in ultrasound examinations to distinguish primary breast cancer from metastasis.

Pathologically analyzing the microcalcifications of breast cancer tumor tissue reveals two main types: dystrophic calcification and metabolic calcification. Dystrophic calcification is predominant in most tumors, while metabolic calcification is rare and often associated with neuroendocrine tumors. Tumor nutrition is primarily facilitated by angiogenesis, driven by vascular endothelial growth factor receptors that promote the formation of irregularly shaped, thin-walled blood vessels lacking a muscular layer. This vascular architecture enhances the tumor’s ability to absorb intravascular nutrients.

In primary breast cancer, monoclonal hyperplasia originates from the ducts or lobules of the breast, leading to the development of nutrient blood vessels comprising both the original breast vasculature and new vessels formed by tumor cells expressing vascular endothelial growth factor receptors. This dual blood supply supports robust tumor growth. The proliferation of tumor parenchyma and stroma, along with stromal fibrosis, can restrict blood flow within the blood vessels of the tumor. This reduced vascular perfusion increases the likelihood of malnutrition and subsequent dystrophic calcifications, resulting in more microcalcifications visible on ultrasound examinations (9).

In metastatic breast cancer, most tumor cells express the vascular endothelial growth factor receptor in tumor blood vessels. The blood supply is relatively small, and metastasis is heterogeneous compared to the breast, often involving a fibrous tissue reaction in the breast stroma. This reaction encases the metastatic lesions, resulting in relatively slow stromal growth, and less noticeable fibrosis, which is not prone to dystrophic calcification.

In this case, after the ultrasound contrast injection, the left cystic nodule of the breast showed slightly higher enhancement in the arterial stage and an enhanced parenchymal phase, with a poorly defined boundary, and regular morphology. The imaging physician considered: left side, breast cystic solid nodules: BI-RADS 4b class, with the possibility of large cystic changes in a fibroadenoma combined with ultrasound contrast. This highlights the limitations of imaging in distinguishing between benign and malignant lesions, underscoring the importance of clinical surgeons being aware of these limitations and emphasizing pathology as the gold standard.

### 3.2 Pathological features

#### 3.2.1 Mirror features

LG-ESS extensively infiltrated the breast stroma with tumor cell islands of irregular size and morphology, exhibiting “lingual” marginal growth (also known as confluent infiltration, the infiltration foci enlarged and widened, and gradually fused into a “tongue shape”) and no stromal response. Under high power, the tumor cells had small cell bodies, scant cytoplasm, and uniform nuclei that ranged from oval to spindle shapes. The cells were either non-atypical or only slightly atypical, with low nuclear division (generally <5/10 HPF) (10). Two of the four patients were LG-ESS, showing consistent morphological features.

HG-ESS tumors can exhibit expansive, osmotic, or infiltrative growth, with multiple infiltration patterns often observed. They typically show infiltration of lymphatic vessels, an active mitotic count, and necrosis. Two of the four patients were HG-ESS, characterized by medium- to highly isoplastic tumor cells, large nuclei with deep staining, and numerous nuclear divisions. However, all patients lacked necrosis and maladaptive epithelial components.

#### 3.2.2 IHC

In two patients with LG-ESS with breast metastasis, the IHC results were as follows: vimentin (+ +), cyclin D1 (+), CD10 (+++), CK (+, locally scattered minority), PR (+/-, partially scattered minority), ER (-), Ki-67 (+, ~40%), H-Caldesmon (-), CD163 (-), CD34 (-), CK5/6 (-), Desmin (-), HMB45 (-), p63 (-), and STAT 6 (-). In two patients with HG-ESS with breast metastasis, both tumor cells showed diffuse positive staining for CD10 and cyclin D1. One patient showed positive staining for CKAE1/AE3 (diffuse +), CKCAM 5.2 (focal +), and p63 (focal +), and negative expression for ER, PR, S100, CD34, Desmin, and ERG. In the other patient, IHC showed positive expression of CKIT, β-catenin, and MDM2. The S100 and p63 were both weakly and locally positive. Desmin, MSA, AE1/AE3, CK5/6, CD34, HMB45, and ERG were all negative. Thus, we concluded that cyclin D1 and CD10 are reliable antibody markers for ESS with metastasis, while CK, PR, S100, and p63 can be either positive or negative.

In this case, the primary lesion of the uterus and the metastatic lesion of the breast showed almost the same immunohistochemical expression(**Table 2**), which showed that the breast mass was caused by the hematogenous tract metastasis after the primary lesion of the uterus.

**Table 2:** Immunohistochemical comparison of primary and metastatic lesions.

	Vimentin	cyclinD1	CD10	CK	S100	SMA	ER	PR	CD34	S100	Ki-67
Primary uterine lesion	+	+	+	+	–	–	–	–	–	–	40%+
Breast metastases	+	+	+	+,locally scattered minority	+, scattered weak	–	–	+/-, partially scattered	–	+, scattered weak	40%+

According to our speculation, the reason for metastasis of low-grade endometrial stromal sarcoma to the breast may be related to the risk factors for tumor metastasis, such as vascular invasion of the tumor observed in the primary tumor lesion of the uterus in this case, resulting in tumor metastasis to the breast through the blood tract. Second, because of the presence of hormone receptors for ER and PR in both the uterus and the breast, low-grade stromal sarcoma of the uterus is ER and PR dependent. This may be why low-grade endometrial stromal sarcoma metastasizes to the breast.

#### 3.2.3 Molecular genetics

In only two patients with HG-ESS, we found a translocation rearrangement of the BCOR gene. Previous literature reportsindicate that HG-ESSs often contain YWHAE-NUTM2A/B, ZC3H7B-BCOR, or BCOR-ITD fusions, and, rarely, EPC1-BCOR, AZF 1-BCORL1, and BRD-8-PHF 1 fusions (11–13). However, about half of LG-ESSs have JAZF1-SUZ12 gene fusion, with a few other cases exhibiting other molecular changes, including JAZF1-PHF1, EPC1-PHF1, and MEAF6-PHF1, among others (14).

### 3.3 Differential diagnosis

ESS with breast metastasis is extremely rare; its tissue morphological characteristics are similar to those of various benign and malignant soft tissue tumors. In clinical diagnosis, if there is no previous history of ESS, secondary diseases can be easily overlooked, presenting significant challenges for diagnostic physicians. The following tumors often need to be differentiated from ESS with breast metastasis:

1. Breast lobar tumor: ESS with breast metastasis often has a lobulated structure with rich stromal cells. LG-ESS and borderline lobar tumor morphologies are similar. The distinguishing point is that lobar tumors, in addition to stromal cell hyperplasia, exhibit lobulated structures, heterogeneous stromal cell distribution, and glandular epithelial hyperplasia. IHC may show expression of CD10, but phyllodes tumors generally express CD34 and B-cell lymphoma 2 (bcl-2) and do not express cyclin D1.
2. Myxoid leiomyosarcoma: This needs to be differentiated from LG-ESS or HG-ESS, mainly due to morphological similarities. IHC is required for differentiation, as leiomyosarcoma often expresses H-Caldesmon and SMA.
3. Myxoid solitary fibrous tumor (SFT): SFT tumor cells are found in both rich and sparse areas, with some typical hemangiopericytoma-like changes and almost no mature fat cells in the tumor. IHC results showed positivity for STAT6, β-catenin, and CD99 in SFT. If necessary, the NAB2-STAT6 fusion gene can be tested in SFT (15).
4. Other mesenchymal tumors: These include inflammatory myofibroblastic tumors, gastrointestinal stromal tumors, malignant melanoma, adenosarcoma, and malignant mixed tumors. Some of these tumors have overlapping morphological features with ESS and require IHC differentiation. The expression of cyclin D1 and CD10 in ESS, along with genetic testing if necessary, can aid in differentiation.

In this case, combined with the clinical history, histopathological characteristics, and IHC results, the diagnosis of LG-ESS with breast metastasis was confirmed.

### 3.4 Treatment and prognosis

When distant metastasis occurs in ESS, the staging standard set by the International Federation of Gynecology and Obstetrics (FIGO) is stage IVb (16). The 2016 National Comprehensive Cancer Network (NCCN) Clinical Practice Guidelines (17) recommend hormonal therapy for patients with LG-ESS IVb and HG-ESS IVb. Some hormone treatments include diprogesterone, medroxyprogesterone, aromatase inhibitors, and gonadotropin-releasing hormone (GnRH) analogs. Postoperative adjuvant therapy for patients with LG-ESS should be individualized based on the conventional pathological results of ER or PR and the expression of CD10 in IHC results. Literature reports suggest that the expression of steroid receptor and aromatase in LG-ESS indicates that adjuvant therapy with progesterone, GnRH analogues, or aromatase inhibitors could be effective (18, 19). However, the benefits of these endocrine therapies are not confirmed without doubt, as there is still no substantial clinical data and insufficient convincing evidence to confirm that hormone therapy is the best treatment option for patients with postoperative lung metastasis of ESS. In summary, the treatment of breast metastasis after ESS still requires exploration through large sample data. Additionally, the prevention of breast metastasis in postoperative patients remains an urgent issue to be addressed.

## 4 Conclusion

ESS with breast metastasis is extremely rare. When the morphological characteristics of ESS with breast metastasis resemble those of certain phyllodes tumors or when the history of uterine ESS is overlooked, it presents significant difficulties and challenges in imaging and pathological diagnosis. Diagnosis should emphasize the combination of clinical history, histomorphology, IHC, and genetic testing when necessary. Accurate diagnosis and rational treatment are crucial for managing this disease. Due to the small number of cases, more evidence-based medical data needs to be accumulated for further analysis to explore the feasibility of adjuvant endocrine therapy, ultimately aiming to develop more effective clinical treatment and follow-up management strategies.

## Data Availability

The original contributions presented in the study are included in the article/supplementary material. Further inquiries can be directed to the corresponding author.
